# Arbutin Alleviates the Liver Injury of *α*-Naphthylisothiocyanate-induced Cholestasis Through Farnesoid X Receptor Activation

**DOI:** 10.3389/fcell.2021.758632

**Published:** 2021-12-02

**Authors:** Peijie Wu, Ling Qiao, Han Yu, Hui Ming, Chao Liu, Wenjun Wu, Baixue Li

**Affiliations:** School of Basic Medical Sciences, Chengdu University of Traditional Chinese Medicine, Chengdu, China

**Keywords:** cholestasis, liver injury, arbutin, farnesoid X receptor, *α*-naphthylisothiocyanate, bile acid metabolism, ursodeoxycholic acid

## Abstract

Cholestasis is a kind of stressful syndrome along with liver toxicity, which has been demonstrated to be related to fibrosis, cirrhosis, even cholangiocellular or hepatocellular carcinomas. Cholestasis usually caused by the dysregulated metabolism of bile acids that possess high cellular toxicity and synthesized by cholesterol in the liver to undergo enterohepatic circulation. In cholestasis, the accumulation of bile acids in the liver causes biliary and hepatocyte injury, oxidative stress, and inflammation. The farnesoid X receptor (FXR) is regarded as a bile acid–activated receptor that regulates a network of genes involved in bile acid metabolism, providing a new therapeutic target to treat cholestatic diseases. Arbutin is a glycosylated hydroquinone isolated from medicinal plants in the genus *Arctostaphylos*, which has a variety of potentially pharmacological properties, such as anti-inflammatory, antihyperlipidemic, antiviral, antihyperglycemic, and antioxidant activity. However, the mechanistic contributions of arbutin to alleviate liver injury of cholestasis, especially its role on bile acid homeostasis via nuclear receptors, have not been fully elucidated. In this study, we demonstrate that arbutin has a protective effect on *α*-naphthylisothiocyanate–induced cholestasis via upregulation of the levels of FXR and downstream enzymes associated with bile acid homeostasis such as Bsep, Ntcp, and Sult2a1, as well as Ugt1a1. Furthermore, the regulation of these functional proteins related to bile acid homeostasis by arbutin could be alleviated by FXR silencing in L-02 cells. In conclusion, a protective effect could be supported by arbutin to alleviate ANIT-induced cholestatic liver toxicity, which was partly through the FXR pathway, suggesting arbutin may be a potential chemical molecule for the cholestatic disease.

## Introduction

Cholestasis, a kind of stressful syndrome induced by hormones, drugs, cytokines, or progressive bile duct destruction or stones, increases the probability of hepatitis, cirrhosis, liver cancer, or other gall-bladder and hepatic diseases due to the excessive accumulation of toxic biliary components like cholesterol, bilirubin, and bile acids (BAs) in the liver and blood (Zollner and Trauner, 2006; Li et al., 2016). Intrahepatic cholestasis has been divided into intrahepatic or drug-induced or inflammatory cholestasis, as well as primary biliary cirrhosis (Zollner and Trauner, 2008; Li et al., 2020b). Upon the stress of toxic biliary, the cholestatic diseases will lead to hepatocyte apoptosis, necrosis, jaundice, hypercholesterolemia, cirrhosis, fibrosis, liver failure, even cholangiocellular or hepatocellular carcinomas, and ultimately life-threatening (Hirschfield et al., 2013; Feldman and Sokol, 2020). Recent studies also reported that the incidence of liver cancer, biliary tract cancer, and several other gastrointestinal tumors is increased when the homeostasis of bile acids metabolism is disrupted (Navaneethan et al., 2021; Ocvirk and O'Keefe, 2021; Rimland et al., 2021; Wu et al., 2021). Disruption of bile acid metabolic homeostasis including dysfunction of BA synthesis, obstruction of the bile duct, and impaired secretion by cholangiocytes will further exacerbate cholestasis (Nakanishi and Saxena, 2015; Keitel et al., 2019). Accordingly, proper recuperation of bile acids is paramount for cholestasis therapy and prevention.

*α*-Naphthylisothiocyanate (ANIT) is commonly applied to induce cholestatic disease *in vivo* and *in vitro* for toxicological studies (Mariotti et al., 2018). Recent studies also suggested the effect of ANIT on hepatocytes by directly destroying bile duct epithelial cells (BECs) (Carino et al., 2020; López-Riera et al., 2020). The excessive accumulation of BAs inside hepatic cells is the primary reason for hepatic damage related to cholestasis. Hepatocytes have two special polarity domains distinguished as basolateral and bile canalicular domains, which are localized with tight junction proteins and specific bile acid (BA) transporters (Li et al., 2016). In general, BAs are produced by cholesterol. Bile canalicular domains containing several canalicular efflux transporters, such as multidrug resistance–associated protein 2 (Mrp 2) and bile salt export pump (Bsep), regulate the transportation of BAs from hepatocytes to the bile canalicular domain for BA generation (Li et al., 2016). While basolateral domains containing organic anion transporter 2 (Oatp2) and Na^+^-dependent taurocholate cotransporter (Ntcp) regulate a primary step in reabsorption of bile acid from portal venous blood to hepatocytes. Besides, other basolateral export transporters, like Mrp-3 and Mrp-4, transport BAs from liver cells to portal blood (Meier and Stieger, 2002; Alrefai and Gill, 2007; Halilbasic et al., 2013; Li et al., 2019). Additionally, a series of bile acid synthetic enzymes containing Cyp8b1 (oxysterol 12*α*-hydroxylase) and cholesterol 7*α*-hydroxylase (Cyp7a1) and metabolizing enzymes like sulfate transferase 2 (SULT2a1) or UDP glucuronosyltransferase 1 (UGT1a1) have also been reported to control the bile acid homeostasis (Thakare et al., 2018). Accordingly, these impaired BA transporters or genes involved in BA metabolisms have been reported to be related to cholestasis. Recently, extensive studies have shown that these aforementioned factors were controlled by specific nuclear receptors (NR) containing the farnesoid X receptor (FXR) signaling pathway. The FXR was reported as the first nuclear receptor of bile acid endogenous ligands, which has been suggested to regulate the bile acid homeostasis and metabolism of cholestasis. Mechanistically, the FXR, heterodimerized with RXR, promoted small heterodimer partner (SHP) binding with BA efflux transporters and enzymes (Stofan and Guo, 2020). Similarly, the FXR-null mice showed increased bile acid–related hepatocyte toxicity and alkaline phosphatase (AP) levels, or decreased expression of BA enzymes and transporters related to cholestasis (Li and Chiang, 2020). Therefore, inhibition of the FXR would aggravate the cholestatic injury; activation of the FXR might be a critical therapeutic target for cholestasis therapy. Ursodeoxycholic acid (UDCA) has been applied for anti-cholestasis drug in clinical trials by the Food and Drug administration (FDA) (Corpechot et al., 2020; Dyson and Jones, 2020). While, more than 40% of patients with primary biliary cholangitis (PBC) cannot be treated adequately and also have serious complications (Dyson and Jones, 2020; Kumar and Kulkarni, 2020). Obeticholic acid (OCA), which could activate the FXR signaling pathway, has been recently approved for PBC by the FDA. However, there are no other proper drugs for cholestatic disease (Kjærgaard et al., 2021). Accordingly, the development of more effective strategies for cholestasis may be imperative. Intriguingly, recent studies implied that some natural products such as picroside and yangonin also have protective effects against cholestasis via the FXR pathway (Dong et al., 2019; Keitel et al., 2019; Li et al., 2020a). Thus, focusing on the FXR pathway may be a promising therapy for liver disease caused by cholestasis.

**FIGURE 1:**
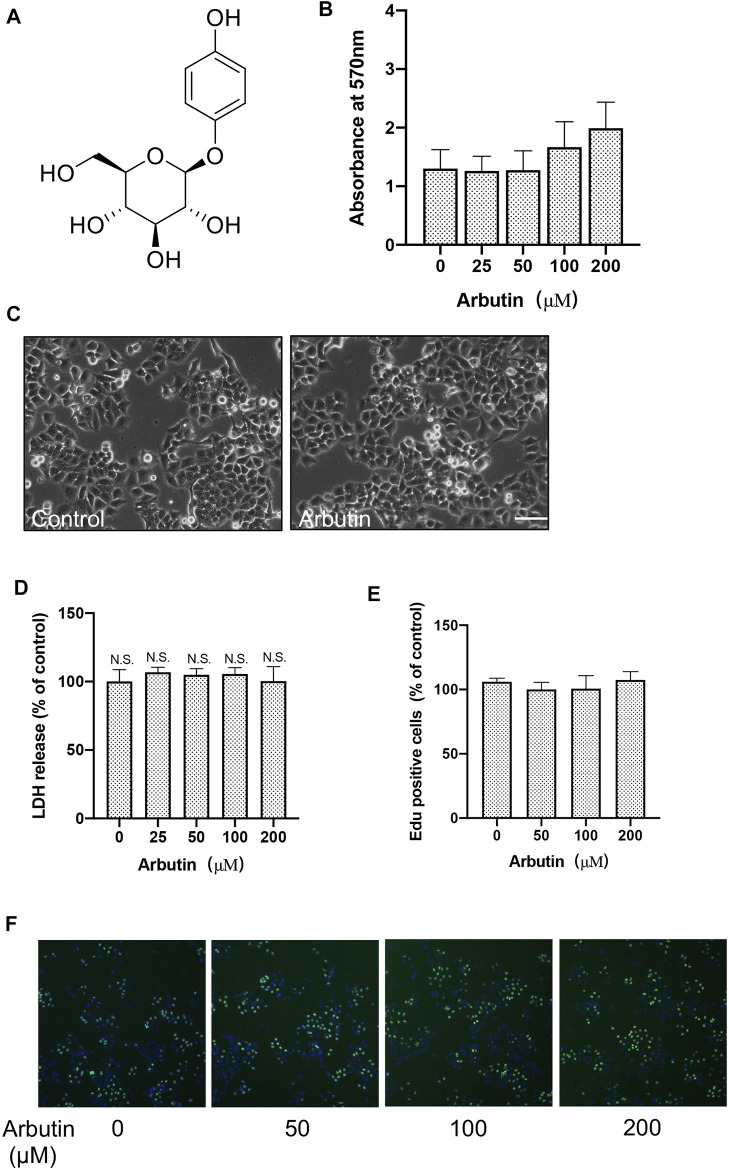
Arbutin has no significant cytotoxic effects on hepatocytes. Effects of arbutin on survival viability and morphology of cells. **(A)** The structure for arbutin. **(B)** Cell viability with or without arbutin treatment was measure by MTT assay. **(C)** Morphologies of cells treated with arbutin for 24 h **(D)** LDH assay was performed with arbutin treatment in L-02 cells. **(E)** Cell proliferation was detected by EdU incorporation assay. **(F)** Proliferation was measured by EdU assay. Representative images are shown.

Arbutin (*β*-d-glucopyranoside of hydroquinone, molecular formula: C_12_H_16_O_7_), a natural hydroquinone glycoside (Figure 1A) present in leaves and fruits of various plants, such as *Arctostaphylos uva-ursi* belonging to Ericaceae and *Saxifragaceae* families, is often used for various skin diseases (Xu et al., 2015; Garcia-Jimenez et al., 2017; Zhou et al., 2019). Leaves or fruits of these plants have been used as traditional medicines for wound healing and urinary tract infections for hundreds or even thousands of years by the aborigines of the American continents and China (Lindpaintner, 1939; Xu et al., 2015; Garcia-Jimenez et al., 2017). And all this time, arbutin has been applied to possess anti-inflammatory, anti-apoptosis, antimicrobial, and antioxidant effects. Even today, arbutin has also been used for the treatment of asthma (Migas and Krauze-Baranowska, 2015; Ortiz-Ruiz et al., 2015; Tang and Chen, 2015). Due to its mildly therapeutic properties, arbutin is also widely used in food, health-care, and cosmetic industries. Previous studies suggested that arbutin may prominently mitigate liver damage caused by CCl_4_ (carbon tetrachloride), radiation, lipopolysaccharides (LPSs), and D-galactosamine (D-GalN) (Mirshahvalad et al., 2016; Nadi et al., 2019; Jurica et al., 2020). But there have been limited research studies on the potential effect of arbutin against cholestatic liver disease and its fundamental mechanisms. In this study, we demonstrated that arbutin can attenuate the injury in ANIT-induced cholestasis, partly *via* FXR pathway activation.

## Materials and Methods

### Chemical Reagents

Arbutin (more than 98% purity) wad supplied by Aladdin (Shanghai, China). ANIT (more than 98% purity) was purchased from Sigma. UDCA was purchased from the Aladdin (Shanghai, China). Ntcp, Bsep, FXR, and Cyp7a1 as well as GAPDH antibody were from Beyotime Biotech (Beyotime, Shanghai, PR China) and Bioss Biotechnology (Bioss, Beijing, China). Goat anti-mouse IgG-HRP or goat anti-rabbit IgG-HRP were purchased from Santa Cruz Biotechnology (CA, United States). Immunofluorescence antibodies of Alexa Fluor were purchased from Invitrogen Life Science (CA, United States). siFXR ((5′-CAA​GTG​ACC​TCG​ACA​ACA​A-3′) was from Qingke Bioscience (Chengdu, China). Kits for determination of alkaline phosphatase (ALP; cat. no. A059-1), alanine transaminase (C0009-2), aspartate transaminase (C010-2), *γ*-glutamyltranspeptidase (*γ*-GT; cat. no. C017-1), total bile acids (TBA; cat. no. E003-1), and direct bilirubin (DBIL; cat. no. C019-2) were obtained from Nanjing Jiancheng. Other chemical reagents were of analytical grade.

### Animals

**FIGURE 2:**
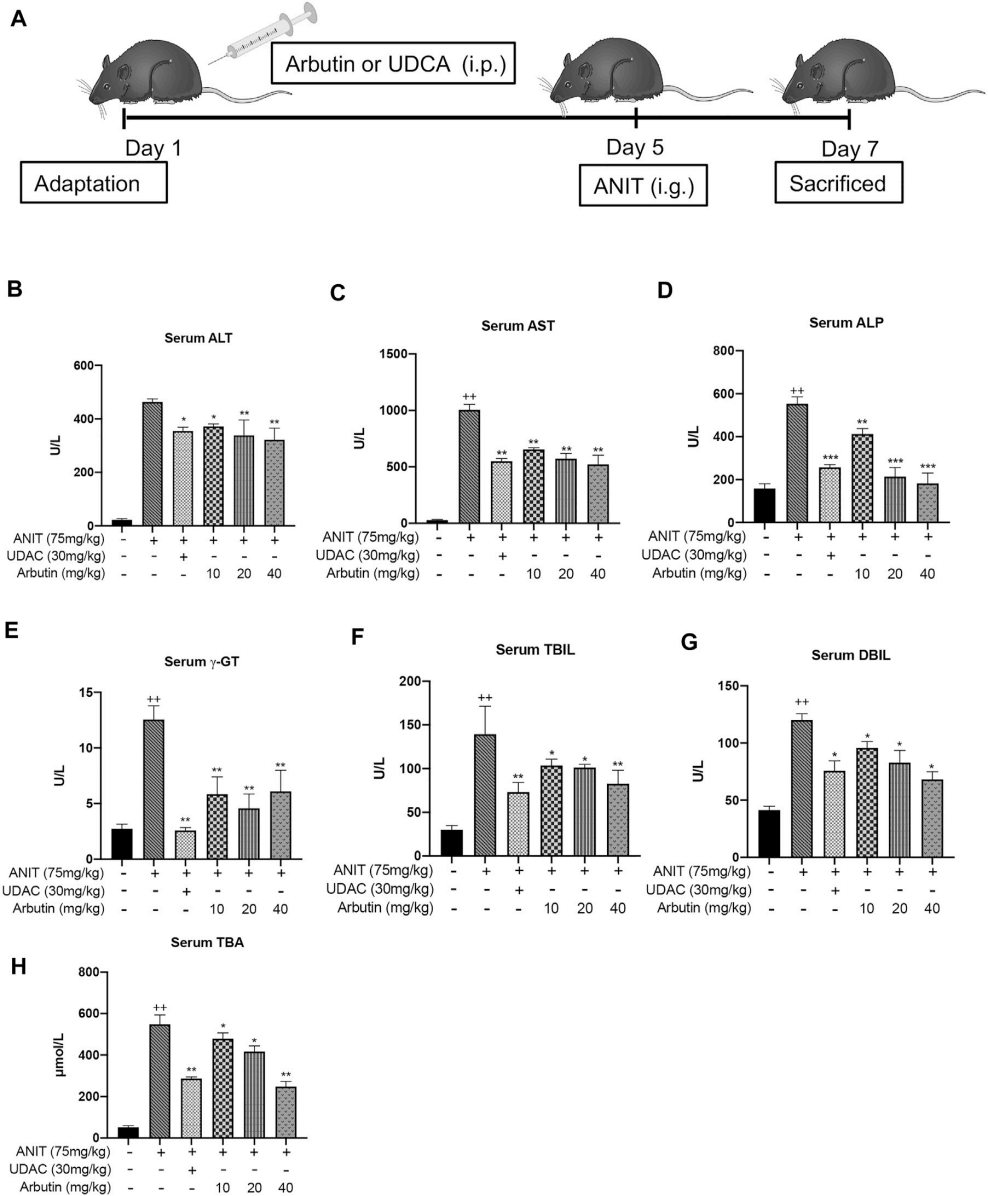
Hepatoprotective effects of arbutin related to cholestatic liver damage induced by ANIT. **(A)** The scheme for the animal experimental design. The serum biochemical indicators levels were elevated **(B–H)**. Data are represented by mean ± SD. (*n* = 3). *p* < 0.05; + *p* < 0.05 vs. control; *p < 0.05 vs. model.

Six- to seven-week-old adult C57BL/6 mice (male) were obtained from Dossy Bioscience (Chengdu, China). The animals were supported on a cycle with 12-hour light and 12-hour dark with temperature (25 ± 1°C) and (46 ± 5%) humidity for 1 week prior to experiments. The C57BL/6 mice were distributed into six groups (eight mice in each group) randomly (Table 1). Chemical drug doses and abidance of exposure were based on previous research studies. Arbutin (10, 20, and 40 mg/kg) or 30 mg/kg UDCA or buffer alone was intraperitoneally (i.p.) administered to mice every day for 1 week. On the fifth experimental day, 3 hours after arbutin, UDCA, or vehicle treatment, mice were intragastrically (i.g.) administered *α*-naphthylisothiocyanate (ANIT) with 75 mg/kg. On the seventh day, all groups were euthanized and fasted to collect blood, bile, and livers for further analysis 4 hours after treatment with arbutin, UDCA, or vehicle buffer. The mice were intragastrically euthanized with 150 mg/kg pentobarbital following the guidelines of the Animal Ethics Committee. The liver, blood, gallbladder, and duodenum were immediately excised after euthanasia and were stored at −80°C. All animal studies were approved by the Animal Ethics Committee of Chengdu University of Traditional Chinese Medicine and performed following the regulations (Figure 2).

**TABLE 1:** Experimental groups and treatment.

Group	Treatment
Control group	Mouse with the same concentrate vehicle buffer
UDCA + ANIT group	Mouse with UDCA every day and ANIT
ANIT group	Mouse with ANIT (75 mg/kg)
Arbutin (low) + ANIT group	Mouse with arbutin (10 mg/kg) every day and ANIT
Arbutin (medium) + ANIT group	Mouse with arbutin (20 mg/kg) every day and ANIT
Arbutin (high) + ANIT group	Mouse with arbutin (40 mg/kg) every day and ANIT

### Histopathology and Serum Biochemistry

After euthanasia, the liver and other tissues were fixed with 4% polyoxymethylene, and the slices were excised from the anterior liver part of the left lobe, embedded in paraffin, and sectioned into 5-µm sections. Each slide was stained with trichrome for hematoxylin and eosin (H&E) and further intuitive liver histological assessment including hepatocellular necrosis, portal inflammation, or hyperplasia. Analysis of the surface areas of the hepatocytes was carried out under a high-resolution microscope (Olympus) with a photographic facility. The samples of blood stored at 4°C for 4 hours were centrifuged for 30 minutes at 12,000 rpm to collect the supernatant. And ALP, *γ*-GT, ALT, TBIL, AST, and TBA of serum were measured using the Automatic Biochemistry Analyzer according to the manufacturers’ instructions.

### Quantitative Analysis of Real-Time PCR

Total RNAs were extracted using TRIzol reagent (Life technologies; no. 15596026) according to the manufacturers’ instructions, and the quantity of total RNA was measured. Reverse transcription was performed to synthesis cDNA with BeyoRT™ commercial Kit from Beyotime (Shanghai, China). As a template, cDNA was used for real-time PCR in triplicate with the SYBR Green Master Mix and specific primers on a Real-Time PCR System (Bio-Rad) (Table 2). GAPDH was normalized as an internal control to evaluate the efficiency.

**TABLE 2:** Sequences of primer for qPCR analysis in this study.

Gene	Forward 5′-3′	Reverse 5′-3′
Ntcp	ATG​GAG​GCG​CAC​AAC​GTA​TC	ACT​ACC​AGA​ATG​ACG​CTG​AGC
Bsep	GGA​CAA​TGA​TGT​GCT​TGT​GG	CAC​ACA​AAG​CCC​CTA​CCA​GT
Oatp1b2	GCA​CTG​CGA​TGG​ATT​CAG​GAT	AGC​TTT​GGT​CGG​TGT​AGC​TTG
MRP2	GTG​TGG​ATT​CCC​TTG​GGC​TTT	CAC​AAC​GAA​CAC​CTG​CTT​GG
Ugt1a1	CTG​AGC​CCT​GCA​TCT​ATC​TG	CCCAGAGGCGTTGACATA
MRP3	ACA​CCG​AGC​CAG​CCA​TAT​AC	TCA​GCT​TCA​CAT​TGC​CTG​TC
Cyp7a1	GAA​CCT​GTA​CAT​GAG​GGA​C	CTT​CTT​CAG​AGG​CTG​CTT​TC
Cyp8b1	GTT​GCA​GCG​TCT​CTT​CCA​T	CCT​TGC​TCC​CTC​AGA​AAC​T
GAPDH	ATG​GAG​AAG​GCT​GGG​GCT​CAC​CT	AGC​CCT​TCC​ACG​ATG​CCA​AAG​TTG​T

### Cell Culture, Cell Viability Assays, and Transient Transfection Experiment

Human hepatocyte (L-02) cells were cultured in DMEM containing 10% FBS, 100 U/ml antibiotics with streptomycin, penicillin, and streptomycin. The viability of L-02 cells was analyzed by MTT experiments. The cells were cultured with indicated concentrations of arbutin (25, 50, 100, and 200 μM) for 24 hours; vehicle groups were cultured with the same final concentration of DMSO (the final concentration must be <0.001). Then, MTT was added to each well to incubate for 3 hours at 37°C. After discarding the supernatant, DMSO was used to dissolve the mixture, and absorbance was measured at a wavelength of 570 nm by thhe Microplate Reader system (BIO-RAD). For transient transfection experiment, L-02 cells were seeded with a proper density for 24-well plates at 7 × 10^5^ cells each well. A control siRNA was used as a negative control. Cells were transfected using Lipofectamine ™ 3000 (Thermo Fisher Scientific). These transfected cells were treated with drugs mentioned before for another 24 hours after transfection. Cell proliferation was detected by the EdU assay kit (RiboBio, C10310) according to the instruction completely. And images were obtained with a fluorescence microscope. For lactate dehydrogenase release (LDH) assay, cell cytotoxicity was assessed by a commercial kit from Beyotime Biotech (Beyotime, Shanghai, PR China). The experiment was carried out in accordance with the supplier’s instructions.

### Protein Analyzed With Western Blot

Total proteins of liver were lysate by RIPA buffer, and the concentration of protein samples were evaluated by a BCA Protein Assay Kit (Thermo Fisher) according to the instructions. Equivalent samples were subjected to SDS-PAGE and then transferred to the polyvinylidene difluoride (PVDF) membranes (Millipore Corporation, MA, United States). After sealing with 5% non-fat milk, the PVDF membranes were washed and co-incubated with specific primary antibodies at 4°C overnight. Subsequently, the second antibody was incubated with the membrane for 1 hour at room temperature. Specific protein–antibody complexes were measured using enhanced chemiluminescence (ECL) reagents and collected with the gel imaging system (ChmiScope, Clinx, China).

### Immunofluorescence

Cells were seeded on the glass cover slides (WHB scientific). After indicated treatment, cells were fixed in 4% formaldehyde, permeabilized by 0.3% Triton X-100, and blocked with 5% BSA. The slides were then incubated with indicated antibodies above at 4°C overnight and Alexa Fluor 488/594 antibodies (1:200 dilution) for 1 hour at room temperature. Nuclei were stained with 1:2000 diluted DAPI solution (Solarbio, C0060) at room temperature for 15 minutes, and the images were captured by using a DM2500 fluorescence microscope (Leica).

### Molecular Docking Study

For molecular docking studies, the AutoDock Tools were used to identify the potential FXR agonists. The 3D structure file of NR1H4 (FXR, code: 4QE6) was downloaded from Protein Data Bank (Kudlinzki et al., 2019). The original structure of NR1H4 was then obtained by AutoDock Tools v1.5.6 to preserve charge and prepare pdbqt file for docking. Then, the hydrogenated SDF structures of arbutin were obtained from PubChem database. And, the structure with the lowest docking energy was measured by minimization. Finally, the resultant protein poses were ranked by scoring, and the image of top-ranked protein pose was further displayed by Discovery Studio Visualizer 2019 and PyMOL v1.8 software.

### Statistical Analysis

All data were presented as mean ± SD. One-way analysis of variance (ANOVA) was used to analyze differences between multiple groups. And non-parametric Mann–Whitney U test and unpaired Student’s t-test were used for two groups with GraphPad Prism software v8. As a threshold, *p* < 0.05 was shown as statistically significant.

## Results

### Arbutin Has No Significant Cytotoxic Effects on Hepatocytes

To investigate the cytotoxic of arbutin on hepatocyte cells, MTT assay of L-02 cells treated with arbutin was performed. The results suggested that 0–200 μM of arbutin for 24 hours showed no obvious proliferative inhibition (Figures 1B,C). Moreover, the IC50 value of arbutin was 618.2 μM (Supplementary Figure S1A); Consistently, EdU incorporation assays and colony formation analysis confirmed that treatment with arbutin has no effects on the proliferation of hepatocyte cells (Figures 1E,F). The results also did not show enhanced cytotoxicity monitored via lactate dehydrogenase (LDH) assay (Figure 1D) Additionally, the serum levels of AST and ALT did not change significantly in mice treated with arbutin 40 mg/kg (data not shown), therefore implicating that arbutin did not exert significant cytotoxicity *in vitro* and *in vivo*. Accordingly, arbutin may have no significant cytotoxic effects on hepatocytes.

### Arbutin Alleviates the Hepatotoxicity and Cholestasis in ANIT-Treated Mice

To evaluate the effects of arbutin on liver injury induced by cholestasis, C57BL/6 mice were treated with ANIT (75 mg/kg) to induce cholestasis. In brief, the vehicle and ANIT groups were treated with vacant solvent. The UDCA treatment with 30 mg/kg and several arbutin treatment groups were, respectively, pretreated with indicated drugs for 5 days. Then, all groups, except the control, were treated with ANIT that resolved in olive oil. Two days later, the serum and liver tissue samples were collected. Our results showed that the levels of ALT and AST in serum and the sensitive blood biochemical index of liver injury were significantly upregulated after ANIT treatment. The levels of TBIL, *γ*-GT, TBA, and ALP in serum, the key biochemical indicators relevant to liver damage, were also elevated by administration with ANIT (Figure 2). Accordingly, these results indicated that intrahepatic cholestatic liver damage had been successfully induced by ANIT. Notably, treatment with UDCA or arbutin visibly reduced the serum ALT and AST levels compared with ANIT treatment. Meanwhile, the levels of serum indicators in the UDCA- and arbutin-treated groups decreased significantly. Moreover, arbutin also reversed the liver injury induced by ANIT (10, 20, and 40 mg/kg arbutin) in an adaptive response way (*p* < 0.01 or <0.05) (Figure 2). Together, these data suggest that arbutin can alleviate the hepatotoxicity in ANIT-induced cholestasis *in vivo*.

### Arbutin Reverses the Morphologic Changes Caused by ANIT in Mice

**FIGURE 3:**
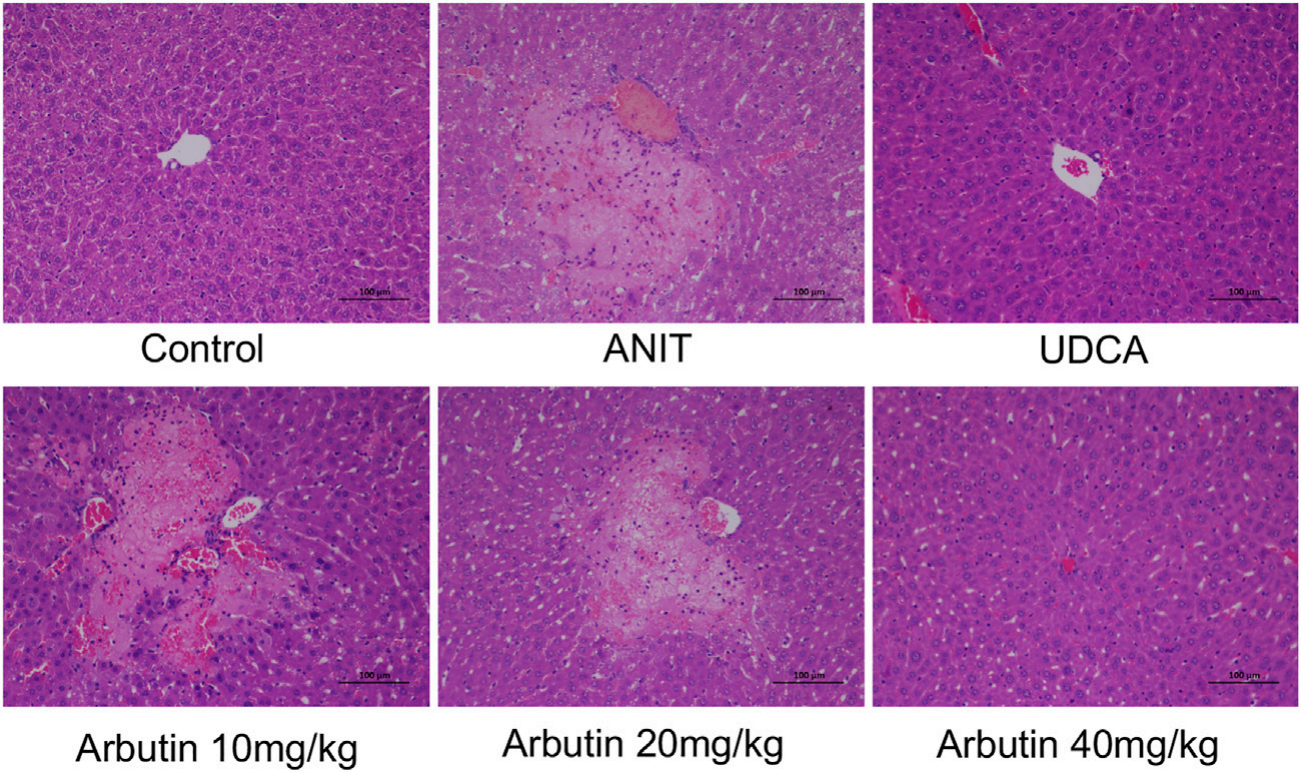
Effects of arbutin on histological or morphologic variations in ANIT-induced cholestasis mouse. (I) Liver tissues by H&E stained (×200).

Morphological and histopathological data of liver were assessed to analyze the hepatoprotective effect of arbutin *in vivo*. The tissues acquired from the ANIT-treated mice displayed visible hepatic necrosis, inflammatory factor infiltration, and edema (Figure 3; Table 3). We also found that ANIT led to a gallbladder filling and a blackened color (data not shown). Congruously, treatment with UDCA and arbutin could significantly attenuate these pathological changes. Arbutin showed a hepatoprotective effect in an adaptive response way (Figure 3).

**TABLE 3:** Effect of arbutin on morphological changes of the liver after ANIT treatment. Data are expressed as the mean of six specimens for each group. −, negative; +, mild (<10 cells).

Group	Dose (mg/kg)	Hepatocyte hydropic degeneration	Hepatocellular necrosis	Inflammatory cell infiltration
Control	–	–	–	–
ANIT	75	+++	+++	+++
UDCA	30	–	–	–
Arbutin (low)	10	++	++	++
Arbutin (medium)	20	+	+	+
Arbutin (high)	40	–	–	–

### Arbutin Alters the Expression of Proteins Related With Bile Acid Homeostasis

**FIGURE 4:**
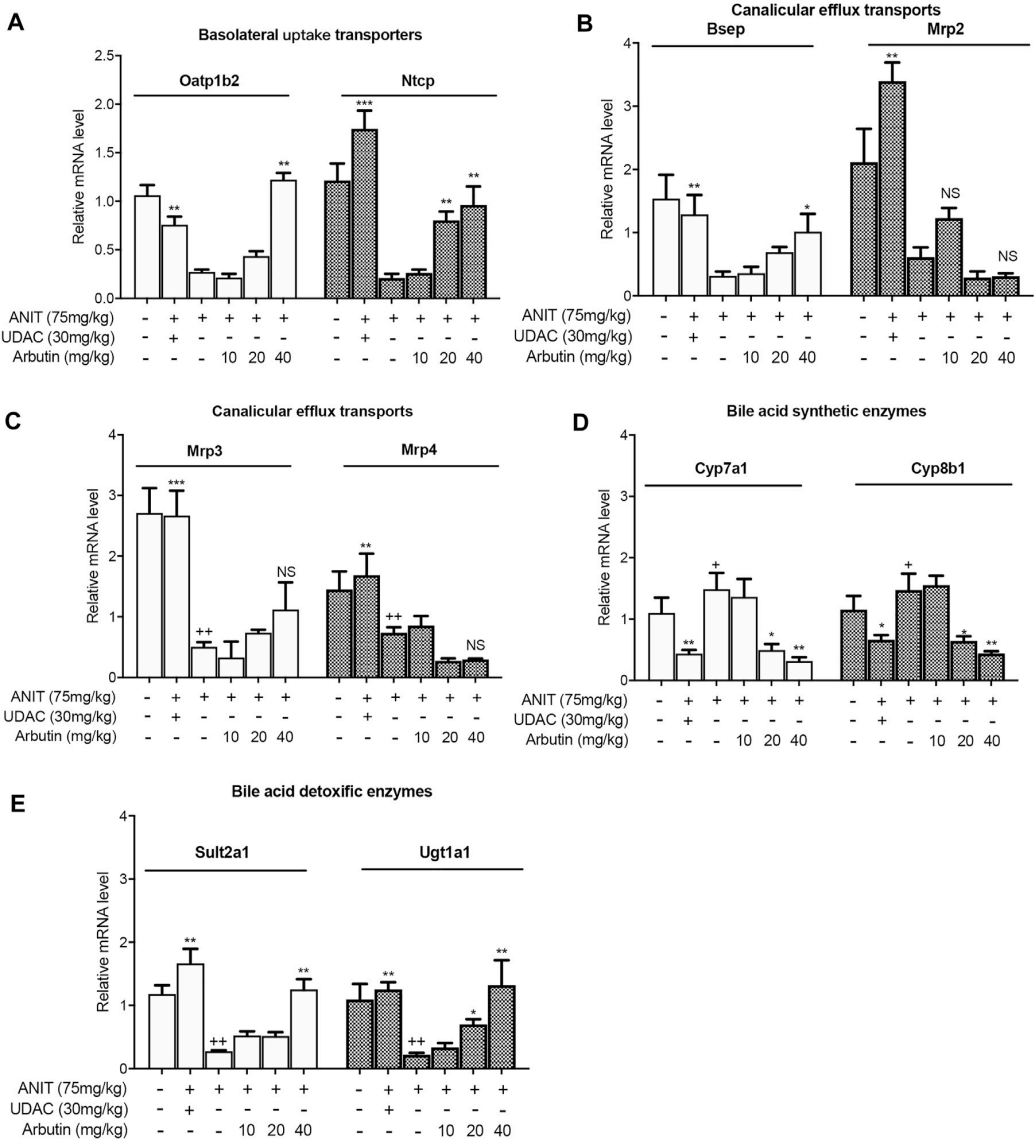
Arbutin changed hepatic genes related to BA metabolism in mouse. Real-time PCR was performed to analyze the levels of genes related to BA metabolism including Oatp1b2, Ntcp, Bsep, Mrp2, Mrp3, Mrp4, Cyp7a1, Cyp8b1, Sult2a1, and Ugt1a1 **(A–E)**. Data are expressed as mean ± SD (*n* = 3). *p* < 0.05; + *p* < 0.05 vs. control; *p < 0.05 vs. model.

In order to investigate the mechanism of arbutin in alleviating liver injury, the genes related to BA metabolism were measured by Western blot or real-time PCR. In our results, ANIT treatment attenuated the transcriptional levels of basolateral uptake transporters, Oatp1b2 as well as Ntcp, which was restored by UDCA or arbutin. The mRNA levels of BSEP and Mrp2, two canalicular efflux transporters, were decreased by ANIT, but arbutin could rescue Bsep, rather than Mrp2 (Figure 4B). For the BA transporters, the mRNA levels of Mrp3 and Mrp4 were reduced in ANIT-treated mice, which could not be affected by arbutin. Furthermore, arbutin reversed the reduced transcriptional levels of Cyp7a1 or Cyp8b1, bile acid synthesis enzymes, caused by ANIT. The levels of Cyp7a1 and Cyp8b1 also decreased when treated with UDCA. Meanwhile, UDCA and arbutin both could restore the expressions of bile acid detoxification enzymes, Sult2a1 as well as Ugt1a1, which are attenuated by ANIT (Figure 4). Altogether, arbutin can reverse the altered several genes expression related to BAs homeostasis.

### Arbutin Activates the FXR-Associated Pathways

As the biosynthesis or transport of bile acid is controlled by the FXR signaling pathway, we analyzed the expression levels of FXR and downstream proteins, Cyp8b1, Bsep, and Ugt1a1 by Western blot. As expected, the suppressed expression of FXR in ANIT-treated mice was restored by UDCA and arbutin.

**FIGURE 5:**
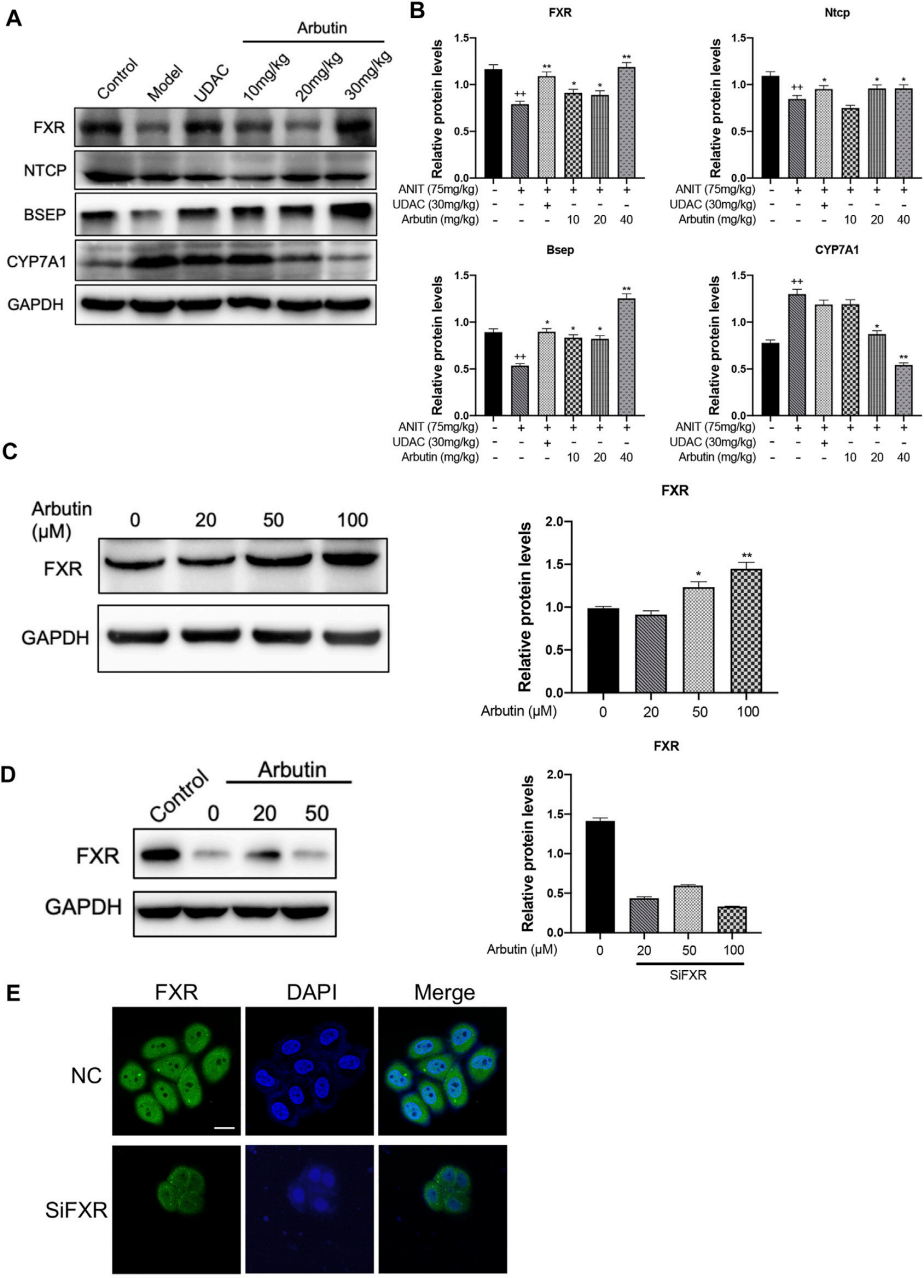
Hepatoprotective effects of arbutin on the FXR signaling pathway in ANIT-induced cholestatic liver disease. **(A)** WB analysis was applied to shown protein levels of FXR, Ntcp, Bsep, and CYP7A1 with treatment indicated. **(B)** The analysis of the indicated proteins. **(C)** WB analysis was used to shown the levels of FXR in L-02 treated with different concentrates of arbutin. **(D)** WB analysis effects of arbutin on the expression of the FXR with or without FXR silence. **(E)** Immunoblotting analysis of FXR protein levels in cells after arbutin was added with or without FXR silence.

We also observed that the downstream biosynthetic enzymes (Cyp8b1, Cyp7a1, and Ugt1a1) and transporters (Bsep, Ntcp, and Oatp1b2) of bile acid could be reversed by arbutin treatment in a dose-dependent way (Figures 5A–C). To further validate whether arbutin could be a potential agonist for FXR, we transfected with siRNA to knockdown the FXR in L-02 cells; we then analyzed the levels of the FXR by Western blot and fluorescence absorbance (Figures 5D,E). Together, these results indicated that the FXR might have participated in arbutin regulated genes involved in BAs metabolism, suggesting arbutin may be a potential and efficient agonist for FXR to exert the hepatoprotection against cholestasis caused by ANIT.

### Molecular Docking Simulations Implicate That Arbutin May Be a Potential Agonist of FXR

**FIGURE 6:**
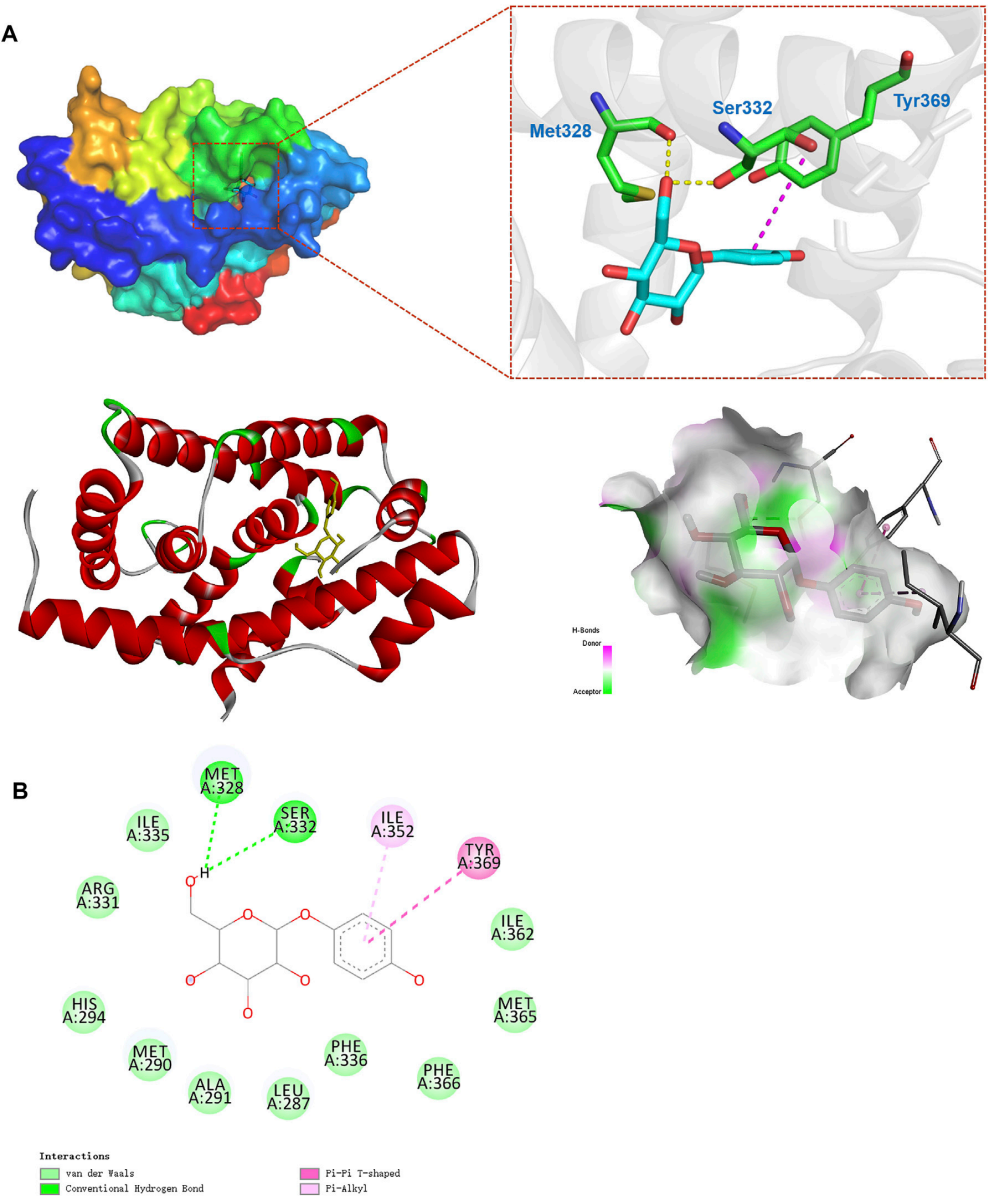
Optimized binding analysis with FXR and arbutin. **(A)** The 3D binding modes between the FXR (PDB code: 4QE6) and arbutin. Electrostatic potential on the FXR molecular surface around the binding site of arbutin and interactions between arbutin and main amino acids in the active site. Hydrogen bonds are shown as dotted lines; **(B)** 2D binding pattern diagram between arbutin and FXR.

**FIGURE 7:**
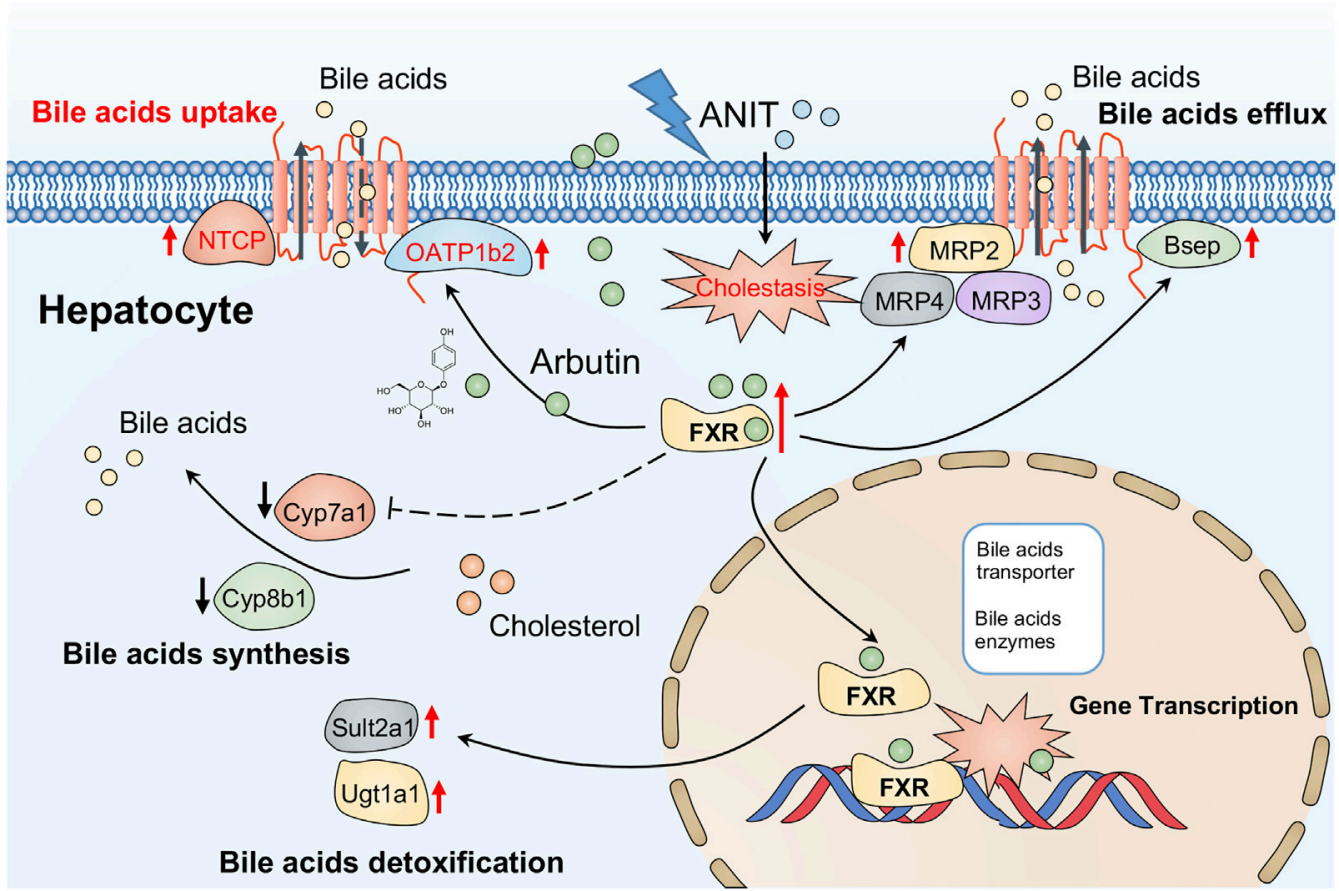
Possible mechanism of arbutin alleviating hepatotoxicity associated with cholestasis induced by ANIT in this study.

To confirm that arbutin has potential to bind to the FXR, molecular docking analysis with arbutin and the residues from the NR1H4 3D structure (code: 4QE6) was performed. Obeticholic acid (OCA) is a well-known potent agonist of the FXR and docked with a score of −11.1 kJ/mol (Supplementary Figure S1B) (Zhang et al., 2020). Similar to the interaction between the FXR and OCA, the energy for binding between arbutin and FXR was −7.4 kJ/mol, implicating that the interaction was stable (Figure 6A). As shown in results, arbutin binds to a hydrophobic pocket consisting of amino acid residues (Met328, Ser332, Ile335, Arg331, His294, Met290, Ala291, Leu287, Phe336, Phe366, Met365, Ile362, Tyr369, Tyr361, and Ile352). Moreover, arbutin formed two essential hydrogen bonds with Met328 and Ser260, and π–π interaction with Tyr369 residues of FXR, which further enhanced the bonding affinity (Figure 6B). Together, the docking model demonstrated that arbutin could bind to the FXR, and arbutin might be a potential FXR agonist (Figure 7).

## Discussion

Cholestatic disease is a common pathological phenomenon in clinics caused by disordered BA homeostasis, leading to toxic gall accumulation, hepatonecrosis, and cirrhosis. ANIT is commonly used to induce cholestatic liver injury *in vivo* and *in vitro* (Mariotti et al., 2018). Recent studies also suggested ANIT-induced effects on hepatocytes, though direct injury caused by ANIT to BECs, which has been demonstrated in many studies (Carino et al., 2020; López-Riera et al., 2020). Currently, UDCA and OCA are commonly used in clinical medicine, but these have a lot of constraints (Floreani and Mangini, 2018; Goldstein and Levy, 2018; Gao et al., 2020). Accordingly, development of appropriate therapeutic medicine for cholestasis is an urgent need. This study demonstrated that arbutin had a visible hepatoprotective effect in the cholestasis model. Consistently, ANIT caused elevated serum levels of ALP, AST, ALT, *γ*-GT, and TBIL related to cholestasis and hepatic function (Carpenter-Deyo et al., 1991; Santamaría et al., 2019; Li et al., 2020a). These disordered serum biochemical indicators conformed to the hepatic cells and structure pattern aberration in the cholestasis mice model. Intriguingly, both arbutin and UDCA could reduce the serum indicators mentioned before. Furthermore, the deterioration of hepatic histology and morphological changes were also alleviated while arbutin and UDCA were administered.

A lot of research studies suggested that maintaining or recovery of the homeostasis of bile acid metabolism via associated transporters and enzymes may be critical for the remission of hepatotoxicity associated with cholestasis. As the homeostasis of bile acids was mainly regulated by BA transporters and synthetic enzymes, ANIT altered the expression of metabolic enzymes for bile acids to aggravate hepatic injury. The expression of Bsep, Mrp2, Ntcp, and Oatp1b2 was remarkably suppressed by ANIT. Several canalicular efflux transporters, such as Mrp2 and Bsep, regulate transportation of bile acid from hepatocytes to bile canalicular domain for bile generation (Li et al., 2016). While Oatp2 and Ntcp play a key role in reabsorption of bile acid from blood to hepatocytes. Besides, the basolateral transporters like Mrp3 and Mrp4 transport BAs from liver cells to portal blood (Keitel et al., 2019). Additionally, a series of metabolizing enzymes like Cyp8b1 and Cyp7a1 are the limiting step enzymes in BA synthesis, while Sult2a1 and Ugt1a1 have also been demonstrated to play key roles in the detoxification of bile acid in the liver (Thakare et al., 2018). Our results suggested arbutin may restore the expression of Bsep, Ntcp, or Oatp1b2 by ANIT, while Mrp2 was slightly altered by arbutin, suggesting that Bsep, Ntcp, and Oatp1b2 may be used for bile acid transportation to protect the liver by arbutin. Mrp3 or Mrp4, as bile acid export transporter, was remarkably reduced in an adaptive responsive way caused by ANIT. While arbutin had a slight effect on Mrp4 or Mrp3, which suggested that these genes may not contribute to this process mediated by arbutin. In the recent studies, we found arbutin played at least three important roles on remission of hepatotoxicity. The first role is upregulaion of bile acid transporters via Bsep and Ntcp. The second is reduction of synthesis of bile acid through Cyp8b1 and Cyp7a1. The last role is regulation of bile acid metabolism by increasing Ugt1a1 and Sult2a1.

Recent evidence has been proved that FXR is a bile acid sensor, which could control series of molecules involved in BA transport, such as Bsep, Mrps, and Ntcp. Furthermore, FXR activation increases the detoxification of bile acid by increasing Sult2a1 as well as indirectly inhibiting the synthesis of bile acid through Cyp7a1 or Cyp8b1 (Xu et al., 2003; Pathak et al., 2017; Al-Aqil et al., 2018; Chambers et al., 2019; Yu et al., 2021). Similar to previous studies, we found that the ANIT significantly inhibited the FXR and its target genes, including Ntcp, Bsep, Cyp8b1, and Ugt1a1 (Jahan and Chiang, 2005; Bertaggia et al., 2017; Petrov et al., 2020). Our results have demonstrated arbutin could reverse the expression of the FXR and downstream genes in the cholestasis mice model, suggesting that arbutin could reduce the synthesis as well as increase transport or detoxification of bile acids by regulating the FXR. Together, we demonstrated that arbutin can attenuate the injury in ANIT-induced cholestasis and systematically investigated the possible involvement of the FXR pathway both *in vitro* and *in vivo*.

## Conclusion

In summary, we demonstrate that arbutin can attenuate the hepatic injury in ANIT-induced cholestasis. The hepatoprotective effect of arbutin may depend on the regulation of bile acid synthetic enzymes and transporters by the activation of the FXR, suggesting that arbutin can be a potential drug to hepatic disease related to cholestasis.

## Data Availability

The original contributions presented in the study are included in the article/Supplementary Material; further inquiries can be directed to the corresponding author.
