# Supplementary figures and images for: Arbutin Alleviates the Liver Injury of α-Naphthylisothiocyanate-induced Cholestasis Through Farnesoid X Receptor Activation
Source: Front Cell Dev Biol. 2021 Dec 2;9:758632. doi: 10.3389/fcell.2021.758632 (PMC8675020; doi:10.3389/fcell.2021.758632)

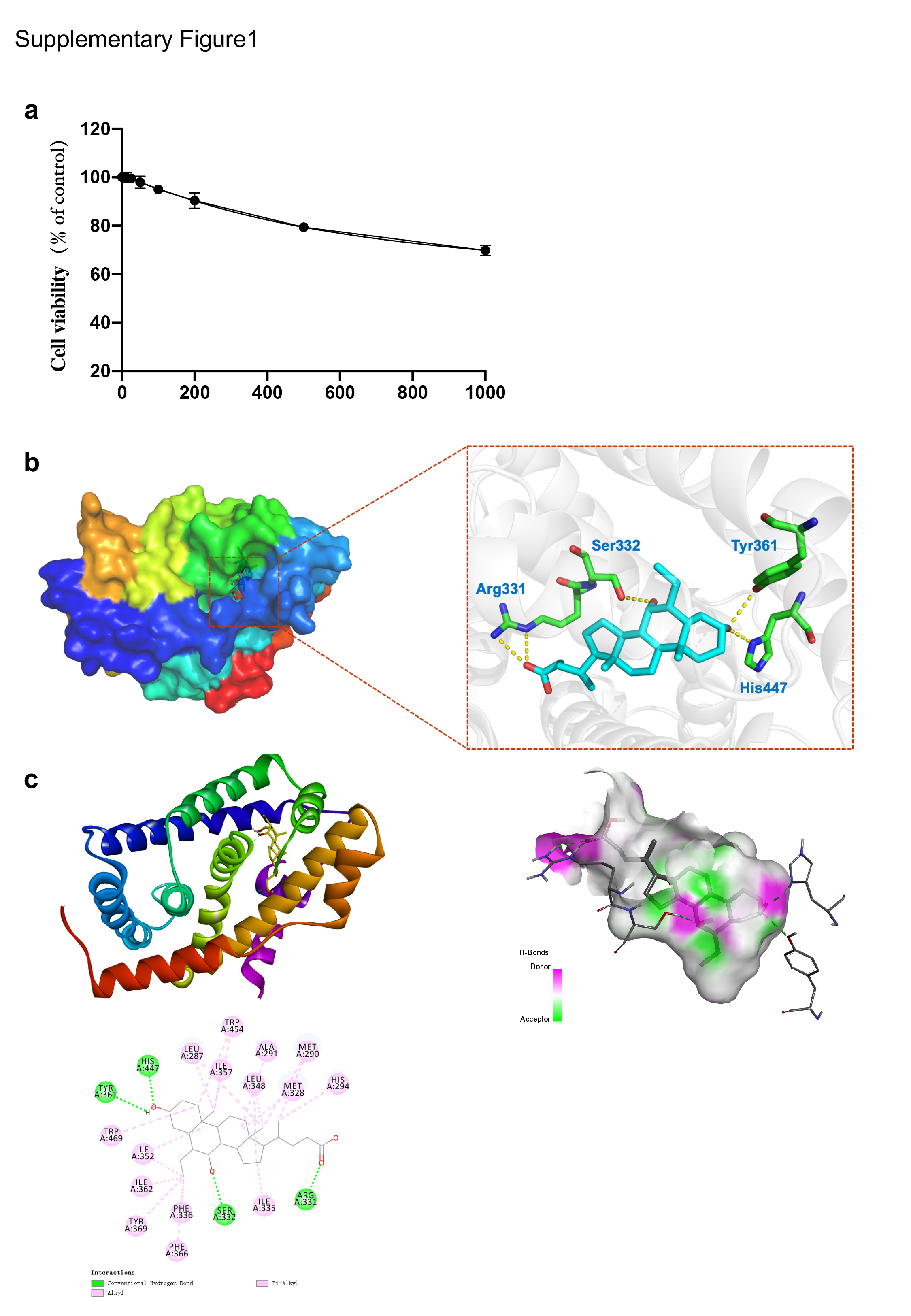

Supplement: Supplementary file 1 [file Image1.JPEG]
